# Supplementary material for: Feasibility of an Adaptive E-Learning Environment to Improve Provider Proficiency in Essential and Sick Newborn Care in Mwanza, Tanzania
Source: medRxiv. 2023 Jul 13:2023.07.11.23292406. Preprint. [Version 1] doi: 10.1101/2023.07.11.23292406 (PMC10370233; doi:10.1101/2023.07.11.23292406)
Supplement: Supplement 1 [file media-1.docx]

Supplementary Materials:


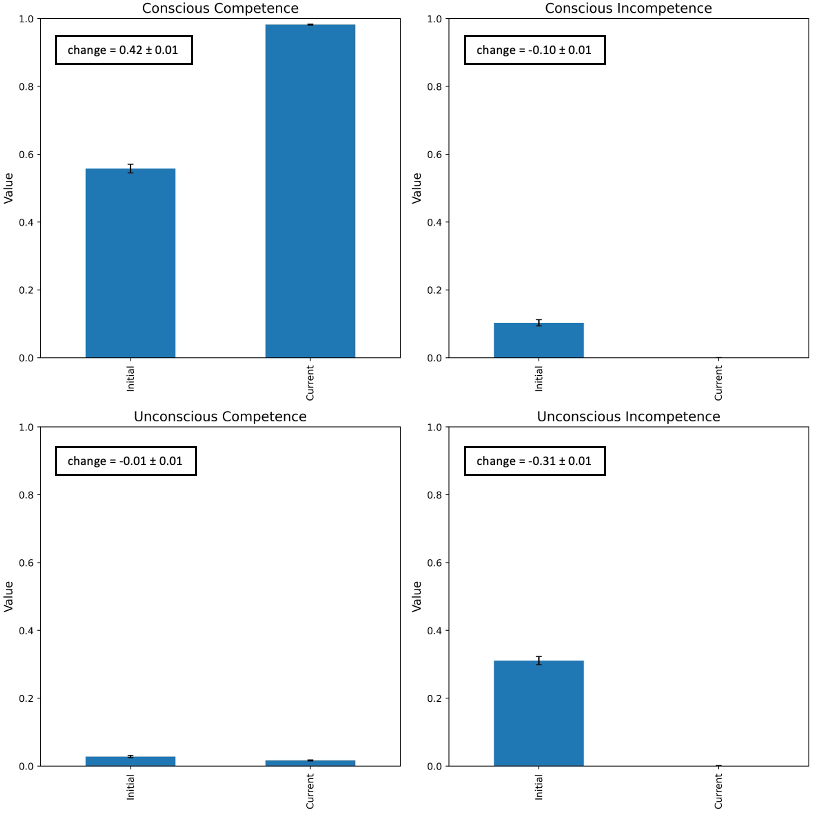

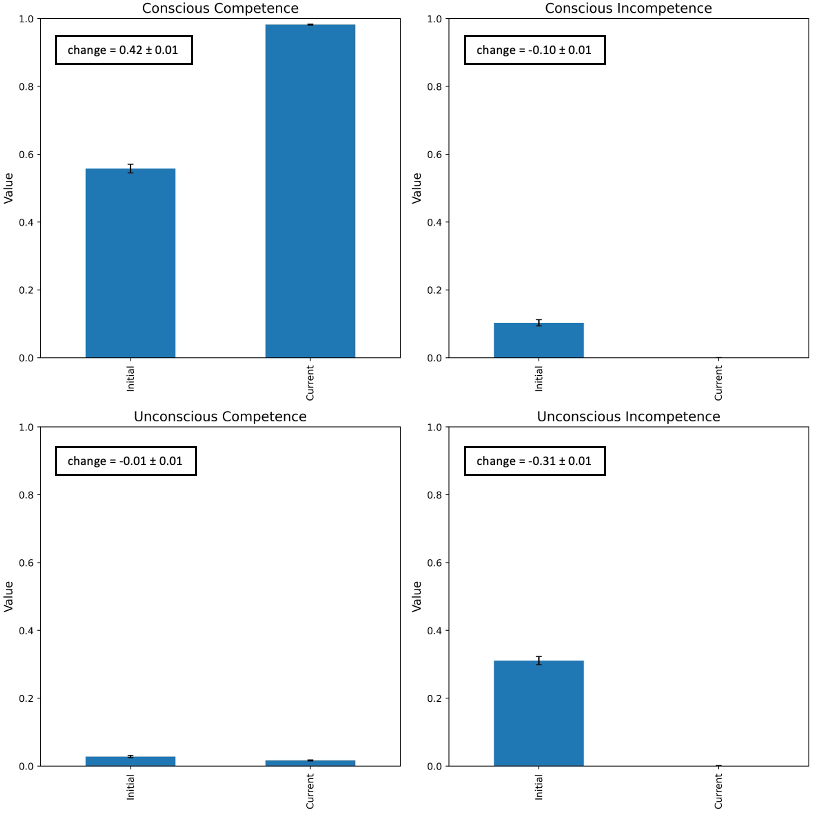


**Figure A. aESNC Efficacy.**


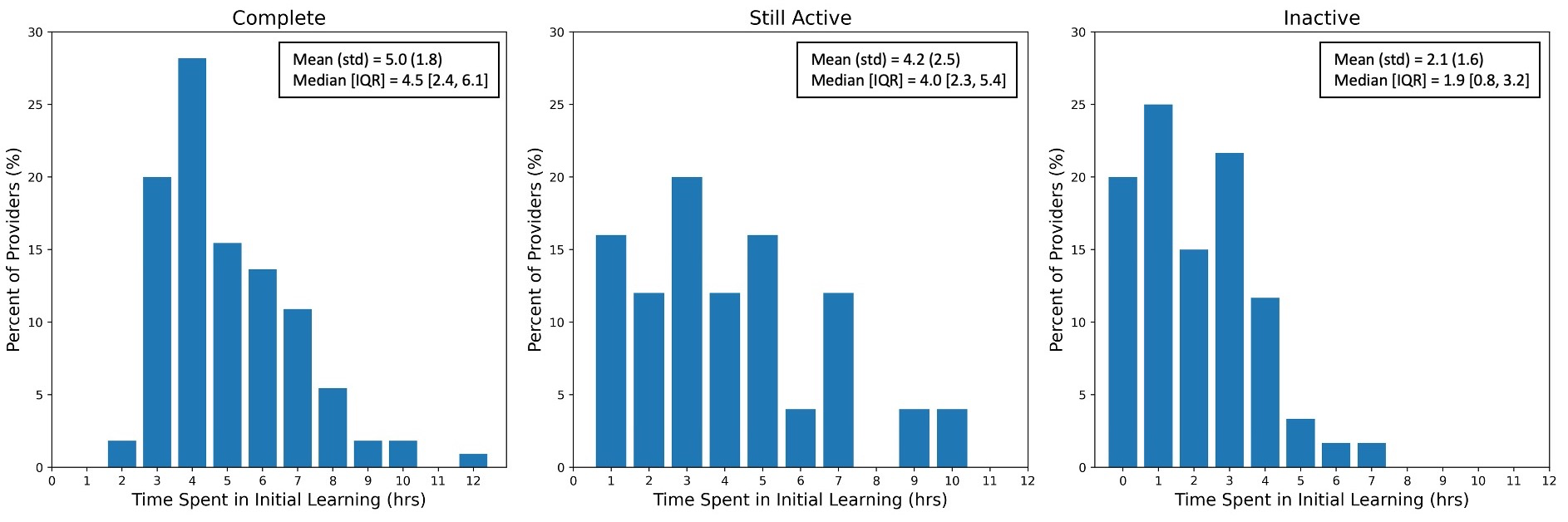


**Figure B1 . Figure B2. Figure B3.**


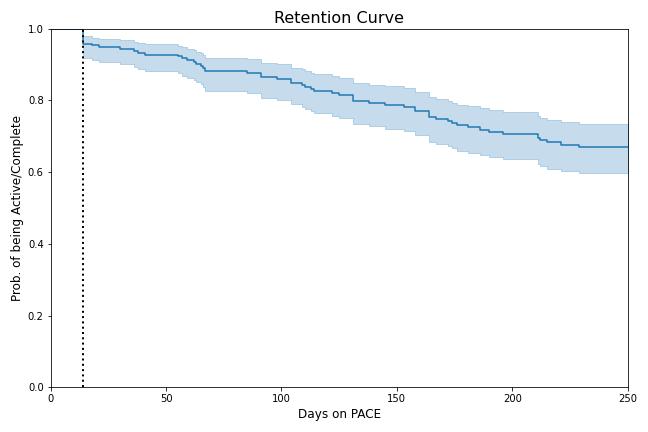


**Figure B4.**


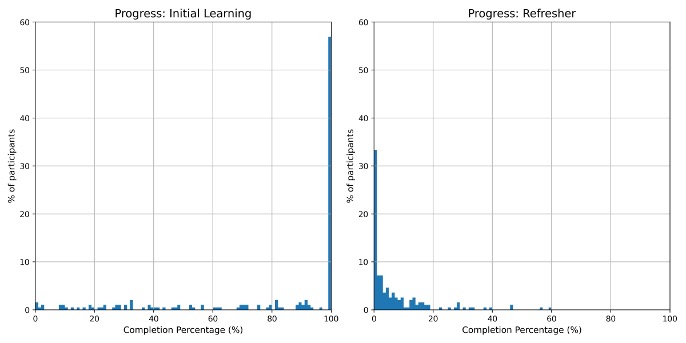

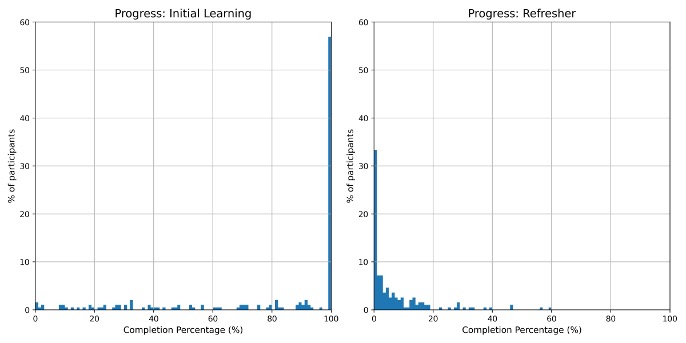


**Figure C1, C2. Progress Distribution**


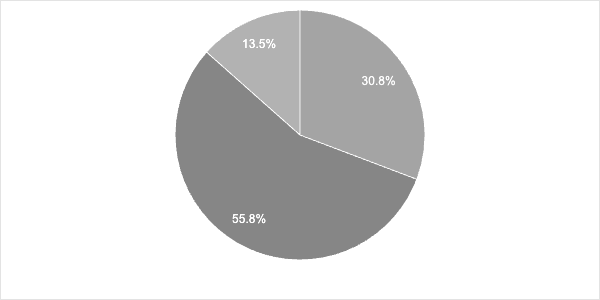


**Figure D1: Nudge types**


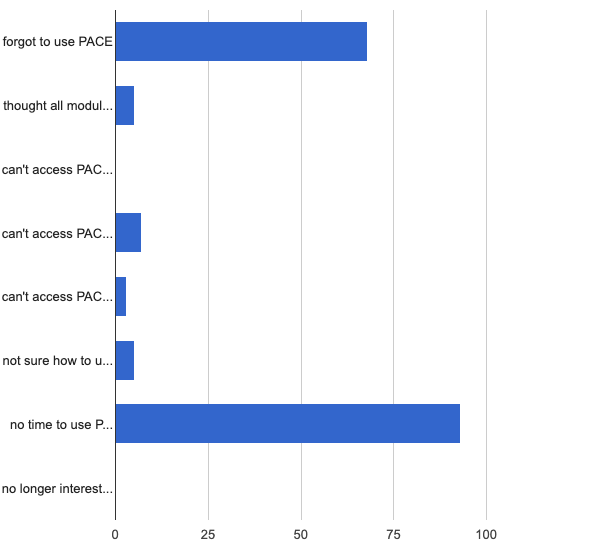


**Figure D2: Reasons for > 30days inactivity**


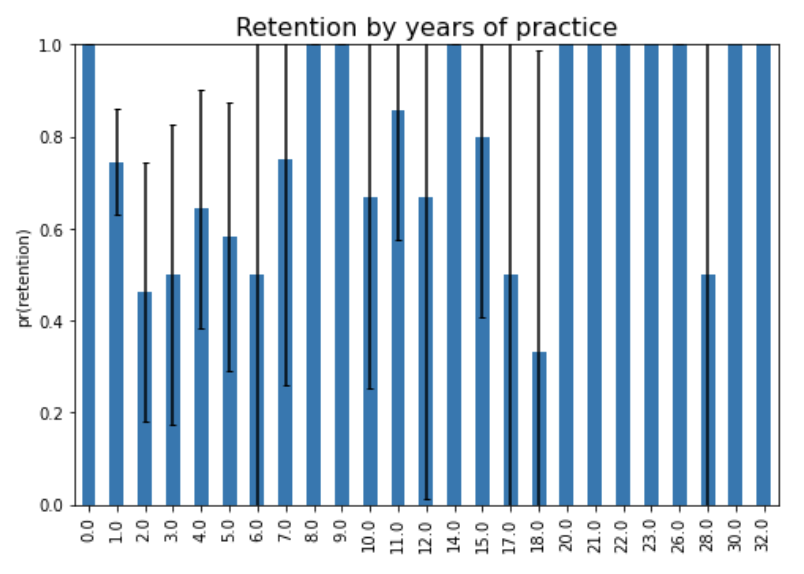


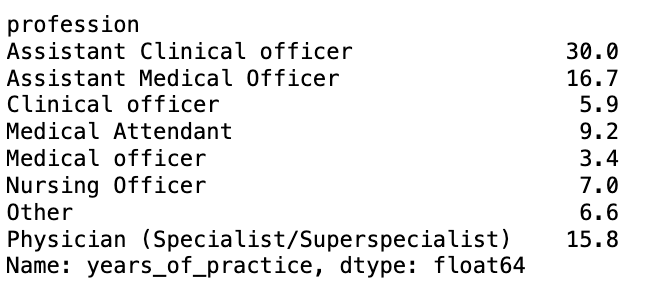


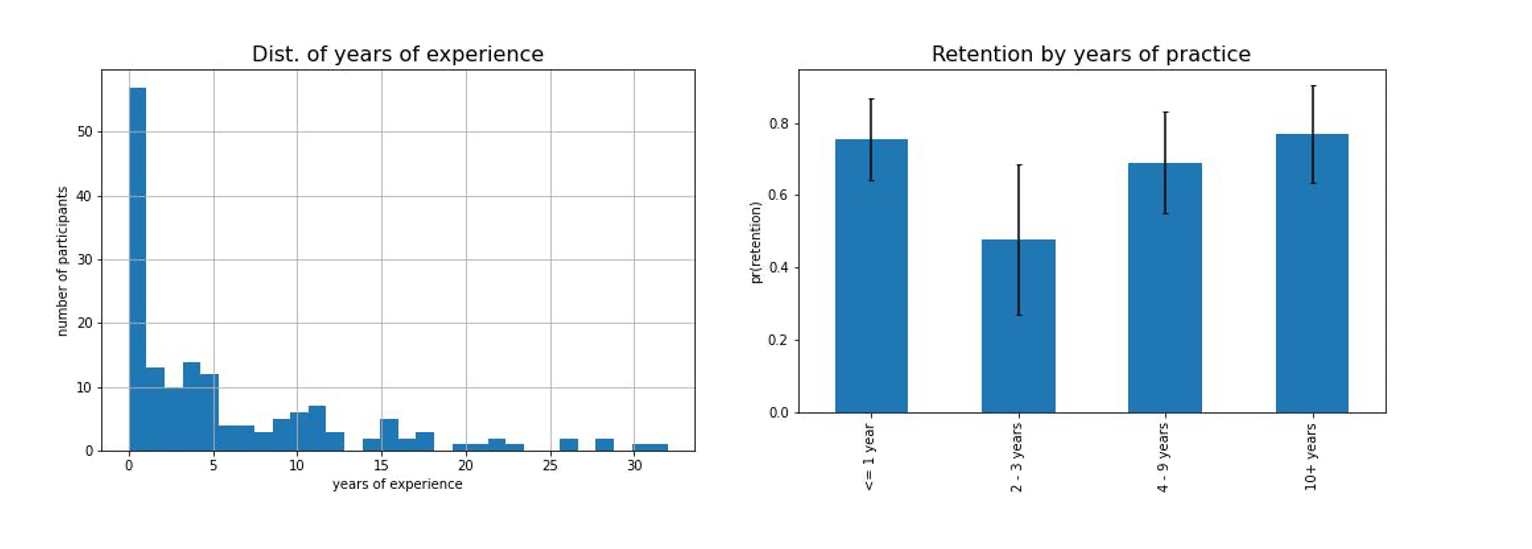


**Figure. Years of experience**

*Providers with 2-3 years of experience have the lowest retention rates.*
